# Vaccine safety surveillance during an mpox outbreak, Germany, June 2022 to February 2024

**DOI:** 10.2807/1560-7917.ES.2026.31.30.2500843

**Published:** 2026-07-30

**Authors:** Doris Oberle, Alexandra Hofmann, Klaus Jansen, Uwe Koppe, Raskit Lachmann, Renz Streit

**Affiliations:** 1 Division of Infectiology, Paul-Ehrlich-Institut, Langen, Germany; 2 Unit HIV/AIDS, STI and Blood-borne Infections, Department of Infectious Disease Epidemiology, Robert Koch Institute, Berlin, Germany; 3 Unit Gastrointestinal Infections, Zoonoses and Tropical Infections, Department of Infectious Disease Epidemiology, Robert Koch Institute, Berlin, Germany; 4 Division of Biomedicines and Diagnostics, Paul-Ehrlich-Institut, Langen, Germany

**Keywords:** mpox, vaccines, adverse events following immunisation, surveillance, monkeypox virus, MPXV, outbreak, vaccine safety

## Abstract

**BACKGROUND:**

In May 2022, first cases of mpox clade IIb were detected in Germany, and MVA-BN vaccine was available from June 2022 onwards. Vaccination has been recommended by the Standing Committee on Vaccination (STIKO) since June 2022 to contain monkeypox virus infection and prevent disease.

**AIM:**

Our primary aim was to evaluate the safety of the mpox vaccine Jynneos (modified Vaccinia Ankara, Bavaria-Nordic, MVA-BN) after the rollout of the vaccination campaign in Germany from June 2022 to February 2024; a secondary aim was to describe mpox cases and vaccinations.

**METHODS:**

To identify cases and persons with adverse events following immunisation (AEFIs), we used existing surveillance systems. A voluntary system was implemented in June 2022 to monitor mpox vaccination. We performed a descriptive analysis of cases, vaccinations, individual case safety reports (ICSRs) and AEFIs.

**RESULTS:**

Until 29 February 2024, 3,807 mpox cases were notified (data retrieved July 2024). According to the voluntary mpox vaccination monitoring, a total of 78,739 mpox vaccine doses of Jynneos were administered. A search of the national AEFI database identified 32 ICSRs, of which seven were serious. A total of 87 AEFIs were reported, most of which were local or systemic reactions that are generally associated with vaccination. The overall adverse event reporting rate adjusted for missing information on vaccine utilisation in one of 16 federal states was 98.7 adverse events per 100,000 doses.

**CONCLUSION:**

The safety profile of the MVA-BN vaccine was in line with the summary of product characteristics. No new safety signals were identified.

**Key public health message**

**What did you want to address in this study and why?**

Mpox is an infectious disease caused by the monkeypox virus and similar to smallpox. Mpox clade IIb started spreading across Europe in 2022. Most diagnoses occurred in men; among cases with known sexual orientation, the vast majority were men who have sex with men. A vaccination campaign started in Germany in June 2022. We wanted to evaluate the safety of the mpox vaccine Jynneos and describe mpox cases and vaccinations.

**What have we learnt from this study?**

At least 78,739 doses of Jynneos were administered in 15 federal states between June 2022 and February 2024. A total of 32 individual case safety reports were submitted, including 87 adverse events following immunisation. In 14 reports, adverse events occurred after the first dose, in six after the second dose and in 12, the dose was unknown. Most frequently reported were headache, itching, diarrhoea, redness at the injection site, fever and hives.

**What are the implications of your findings for public health?**

In Germany the safety profile of Jynneos appears favourable in the vaccinated high-risk population, which is predominantly made up of men who have sex with men, thereby supporting targeted vaccination strategies. However, very rare serious events cannot be ruled out and require ongoing surveillance.

## Introduction

Mpox disease is caused by the monkeypox virus (MPXV), an enveloped DNA virus of the family Poxviridae, genus Orthopoxvirus (OPXV). The virus is related to the classical human smallpox viruses (variola, smallpox) and the cowpox viruses. Two distinct clades of MPXV have been identified: Clade I, previously known as the Congo Basin, Central African clade, and Clade II, the former West African clade. Clade IIb was responsible for the global outbreak that was ongoing at the time of writing [1]. In this outbreak, human-to-human transmission occurred mainly through close skin-to-skin contact, especially during sexual activities among men who have sex with men (MSM).

Monkeypox virus infection can be asymptomatic. However, it may be accompanied by one or more systemic symptoms, such as fever, headache, aching limbs and painful swelling of the lymph nodes. Night sweats are also common, typically presenting alongside skin lesions, with the anal and/or genital areas being the most frequently affected body sites in the German outbreak in 2022 [2]. However, the mpox rash typically appears in a centrifugal pattern, affecting the face, trunk and extremities [3]. Skin manifestations may also be localised to the palms, soles, oral mucosa, conjunctivae and cornea [4]. Gastrointestinal symptoms such as pain, anorexia, diarrhoea, nausea and vomiting and/or liver injury were also reported [5].

In May 2022, the first cases of mpox clade IIb were detected in Germany [2]. Since June 2022, vaccination has been offered in Germany (estimated population size in 2022: 82.7 million [6]) in order to contain MPXV infection and prevent disease. This was supported by intensive information and vaccination campaigns addressing sexually active MSM, set up in close collaboration with national and federal community organisations. Until August 2023, the vaccine available in Germany was Jynneos which contains a replication-deficient live modified form of the vaccinia virus called ‘vaccinia Ankara’ (modified Vaccinia Virus Ankara – Bavarian Nordic, MVA-BN). It was authorised by the United States (US) Food and Drug Administration (FDA) in 2019 for prevention of smallpox or monkeypox in adults aged ≥ 18 years determined to be at high risk of infection with the causing viruses [7] and was sourced from the US.

The MVA-BN vaccine Imvanex was authorised in the European Union (EU) for the prevention of smallpox in adults on 31 July 2013. It is almost identical to Jynneos, with only minor differences in the manufacturing process and quality specifications. On 21 July 2022, the Committee for Medicinal Products for Human Use (CHMP) at the European Medicines Agency (EMA) adopted a positive opinion recommending an amendment to the terms of the marketing authorisation for the medicinal product Imvanex to add a new therapeutic indication for the active immunisation against monkeypox and disease caused by vaccinia virus [8]. The Commission’s decision was issued on 22 July 2022 [9]. Since then, the full indication for Imvanex therefore has been as follows: “*Active immunisation against smallpox, monkeypox and disease caused by vaccinia virus in adults*”.

Jynneos and Imvanex are administered subcutaneously, preferably in the upper arm. Persons who have not previously been vaccinated against smallpox, mpox or the disease caused by vaccinia virus should receive two 0.5 mL doses, with the second dose given at least 28 days after the first. If a booster dose is considered necessary in previously vaccinated persons, a single 0.5 ml dose should be given. Immunocompromised individuals who have previously been vaccinated against smallpox should receive two vaccinations.

Since June 2022, the Standing Committee on Vaccination (STIKO) has recommended vaccination with Imvanex or Jynneos (MVA-BN) for post-exposure prophylaxis (PEP) and for persons at increased risk; this primarily includes MSM who frequently change partners [10].

A systematic review has analysed data on the effectiveness of the smallpox vaccine in preventing MPXV infection pooled from 11 studies including 13,505 vaccinated and 19,786 unvaccinated participants; it showed that the risk of mpox infection was reduced by 54%, and a subgroup analysis for Europe showed a risk reduction by 60% [11]. Adverse events following immunisation (AEFIs) with mpox vaccines are most often local or systemic reactions that are known to occur after immunisation: In a pooled analysis of studies evaluating the safety of smallpox vaccine for the prevention of MPXV infection, the incidence was 56.3% for local erythema, 55.1% for local swelling, 49.7% for local induration, 47.8% for local itching and 39.2% for local pain after vaccination. The incidence of vomiting, shivering, headache, rash, myalgia and fatigue ranged from 0.9% to 19% [11].

Although safety information is available from the pooled analysis of safety studies, is also necessary to report on safety surveillance during specific outbreaks. Our primary aim was to evaluate the safety of the mpox vaccine Jynneos after the rollout of the vaccination campaign in Germany from June 2022 to February 2024. A secondary aim was to describe mpox cases and vaccinations with Jynneos. Germany is a nation with a comparably high number of doses administered.

## Methods

### Mpox case surveillance

In Germany, according to the Protection Against Infection Act (IfSG) [12], physicians and laboratories must report to the local public health authority (LPHA) suspected illness, illness and death caused by orthopox viruses by name of the sick or deceased person. Direct or indirect evidence of orthopox viruses must be reported by name of the sick or deceased person to the LPHA if the evidence points to an acute infection. The reports must be submitted to the LPHA no later than 24 h after the information has been obtained. After a case has been reported, the LPHA also carries out investigations, such as contact tracing. If a case fulfils the reference case definition created by the Robert Koch Institute (RKI) [13], which we append in full in the Supplement, it is electronically submitted in anonymised form via the Federal State Authority to the Robert Koch Institute (RKI), the German Institute for Public Health. 

We retrieved data on mpox cases from SURVSTAT@RKI 2.0 (https://survstat.rki.de). Information on mpox vaccination was not included there.

### Mpox vaccination monitoring

The RKI set up a voluntary mpox vaccination monitoring system immediately after cases of mpox clade IIb occurred in Germany [14]. Jynneos was procured centrally at national level. The State distributed the vaccine among the federal states. The decision on how many vaccine doses each federal state should receive was made by the Federal Ministry of Health and the federal states in consultation with the German Centre for Pandemic Vaccines and Therapeutics on the basis of the estimated population sizes of MSM potentially susceptible for mpox. The federal states authorised specific centres (such as public health offices, specialised medical practices and outpatient clinics) to vaccinate persons according to the STIKO recommendation. Imvanex was procured and distributed through standard channels, i.e. purchasing vaccine doses from pharmaceutical companies via pharmacies and was therefore not covered by the mpox vaccination monitoring. It was voluntary for the federal states to participate in the vaccination monitoring. 

In an initial phase (June 2022 to August 2022), the vaccination centres reported data, on single case basis, on the federal state, the type of vaccination centre, month and year of vaccination, batch number and type of dose (first or second dose) as well as anonymised information of the vaccinated person on gender, age group, prior pox vaccination in childhood and reason for vaccination (PEP, private indication, occupational indication), and reported the data to their federal state authority. The federal state authorities forwarded the data to the RKI. Later, a digital system was set up and from September 2022, the vaccination centres could submit their data via an anonymous online form directly to the RKI which analysed the data and reported the number of vaccinated cases to the federal states and to the public in aggregated monthly reports. Mpox vaccination monitoring was discontinued at the end of February 2024 after Imvanex became regularly available in Germany besides the initial central procurement of Jynneos.

### Spontaneous adverse events reporting system

The Paul-Ehrlich-Institut (PEI), the Federal Institute for Vaccines and Biomedicines, receives individual case safety reports (ICSR) of AEFIs with vaccines (Imvanex, Jynneos, others) via the LPHAs in accordance with IfSG [12]. Physicians are legally obliged to report AEFIs, i.e. health complaints that go beyond the usual extent of a vaccination reaction and are not obviously due to other causes, by name of the vaccinee to the LPHA, which in turn reports them immediately and in pseudonymised form (i.e. without providing the vaccinee's name and address) to the PEI. In addition, the PEI receives reports from pharmacists (Drug Commission of German Pharmacists) and physicians (Drug Commission of the German Medical Association), as pharmacists and physicians have a professional obligation to report adverse events following intake of any drug to their respective Associations. 

In addition, vaccinated persons or their relatives can report directly to the PEI. Reports can be made by post, e-mail, telephone or electronically via the PEI’s reporting portal (www.nebenwirkungen.bund.de). The PEI combines identical reports from different sources into one case.

All received ICSR are pseudonymised and stored in the PEI’s adverse drug reaction database (Vigilance*ONE* Ultimate, version 4.8.1.4, 2003-2021 PharmApp Solutions GmbH, Erkrath, Germany; http://www.PharmApp.de). According to the German Medicinal Products Act, the PEI is obliged to report AEFI electronically at certain intervals in an internationally standardised format and pseudonymised to the joint EudraVigilance database at the EMA, to which every regulatory authority in the EU has access. 

According to the German Medicinal Products Act (§63c) [15], marketing authorisation holders are obliged to report to the European adverse drug reaction database EudraVigilance. Reports from Germany are then forwarded to the PEI.

The spontaneous reporting for mpox AEFI follows the same rules as for all other vaccines administered in Germany. The ICSRs were coded by trained data entry staff according to the Medical Dictionary for Regulatory Activities (MedDRA) [16] in lowest level terms, which provide maximum specificity. In the MedDRA terminology, the selection of a lowest level term results in automatic assignment of grouping terms higher in the hierarchy: preferred terms (PTs), high level terms, high level group terms, and system organ classes.

### Individual case safety reports retrieval and plausibility checks

A search for ICSRs related to Jynneos was performed in the Vigilance*ONE* Ultimate database on 22 July 2024, including AEFIs with a start date up to 29 February 2024. We excluded ICSRs on vaccination failure. Retrieved ICSRs underwent an in-house review by trained medical staff of the PEI focussing on the details of the reported case. Inconsistencies regarding medical history, the administered vaccine, concurrent drugs or therapies, time to symptoms onset, information on AEFIs, clinical course and outcomes were considered. In case of inconsistencies or missing information (e.g. on batch number), the reporting person was contacted, and follow-up information was requested.

### Seriousness

The seriousness of ICSRs was determined according to ICH Topic E2A, Clinical Safety Data Management: Definitions and Standards for Expedited Reporting: Reference Number: CPMP/ICH/377/95 [17]. The details of this classification are appended in the Supplement.

### Causality assessment

We used the World Health Organization (WHO) algorithm and classification to assess the association between an AEFI and mpox vaccination for all ICSRs [18]. Levels of causality assessment were: consistent with a causal association with immunisation, indeterminate, inconsistent with a causal association with immunisation, unclassifiable. The criteria for this classification are listed in the Supplement.

### Statistical analyses

Data on mpox cases notified according to IfSG and the mpox vaccination monitoring were analysed separately. We performed a descriptive statistical analysis. We calculated absolute and relative frequencies for qualitative variables, median and interquartile ranges for quantitative variables. The number of mpox vaccinations administered obtained via the mpox vaccination monitoring was used to estimate the AEFI reporting rates (number of events per 100,000 vaccine doses administered). To adjust the number of administered vaccine doses for the missing data from the federal state that did not participate in the voluntary mpox immunisation monitoring (15 of 16 states participated), these data were estimated using the number of doses delivered to this state(s) multiplied by the mean proportion of the number of doses administered from the stock in the participating federal states and added to the number of vaccinations reported through the voluntary mpox vaccination monitoring. The adjusted number of administered mpox vaccinations was used as denominator to calculate the adjusted ICSR rate, the adjusted serious ICSR rate and the adjusted adverse event reporting rate. Statistical analysis was performed using Statistical Analysis System (SAS Institute Inc., US, version 9.4).

## Results

### Mpox cases

A total of 3,807 mpox cases were notified in Germany from May 2022 to February 2024 (data retrieved on 22 July 2024). In 2022, the German population included 82.7 million individuals. Calculating the rate per 100,000 person-years during the observation period (May 2022 to February 2024) gives an incidence rate of 2.5 mpox cases per 100,000 person-years.

The majority of mpox cases were reported between May and September 2022 (Figure). The characteristics of the mpox cases are summarised in Table 1. Almost all patients (99.4%) were male, and mainly in the age groups 30–39 years (41.3%) and 40–49 years (26.8%).

**Figure:**
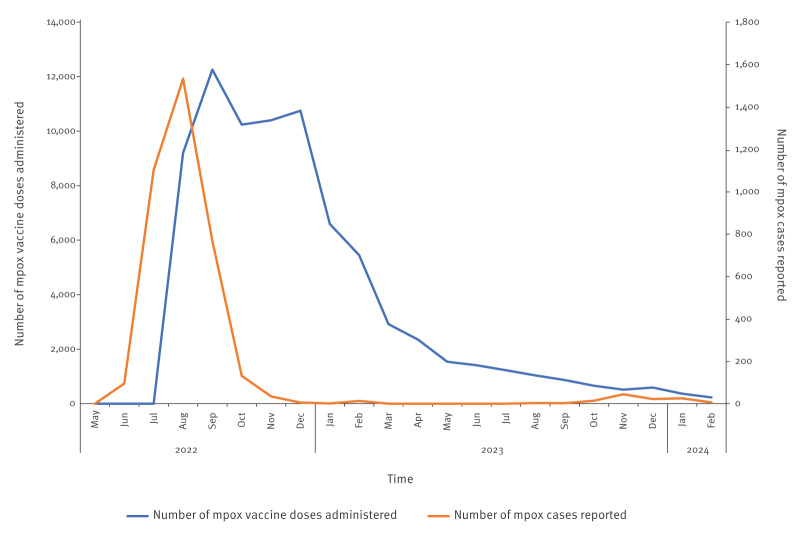
Mpox cases (n = 3,807) reported to the Robert Koch Institute and mpox vaccine doses administered^a^ (n = 78,739), Germany, May 2022–February 2024

^a^ Not adjusted for the non-participating federal state (15 of 16 states participated).

**Table 1:** Characteristic features of mpox cases, Germany, May 2022–February 2024 (n = 3,807)

Characteristic feature	n	%
Gender		
Men	3,785	99.4
Women	20	0.5
Diverse	0	0.0
Undetermined/unknown	0	0.0
NA	2	0.1
Age (years)		
< 18	7	0.2
18–29	655	17.2
30–39	1,572	41.3
40–49	1,020	26.8
50–59	432	11.3
≥ 60	121	3.2
Unknown	0	0.0

NA: not available.

### Mpox vaccinations

All but one of the German federal states participated in the voluntary mpox vaccination monitoring. The participating vaccination centres covered about 84.2% of the German population. Doses for the non-participating state were estimated as described in Methods.

A total of 78,739 doses of mpox vaccine were reported to be administered between June 2022 and February 2024 (data status: 25 April 2024) (Figure), with the majority administered between June 2022 and April 2023. Vaccine utilisation (i.e. vaccine doses administered/vaccine doses delivered) varied between the participating federal states from 12.5% to 60.5% with a mean vaccine utilisation of 35.5%.

The characteristics of the vaccinations are described in Table 2. There were 30,105 complete vaccination series, if we assume that every reported second vaccination was preceded by a reported first vaccination.

**Table 2:** Characteristics of administered mpox vaccination doses^a^, Germany, June 2022–February 2024 (n = 78,739)

Characteristic feature	n	%
Gender of vaccine recipient		
Men	76,475	97.1
Women	693	0.9
Diverse	345	0.4
Undetermined/unknown	60	0.1
NA	1,166	1.5
Age (years) of vaccine recipient		
< 18	24	<0.1
18–29	12,986	16.5
30–39	27,173	34.5
40–49	20,015	25.4
50–59	13,009	16.5
≥60	4,113	5.2
Unknown	1,419	1.8
Vaccination setting		
Medical practice	62,537	79.4
Local public health authorities	5,781	7.3
University hospital/outpatient clinic	8,868	11.3
Community-based voluntary counselling and testing centres	1,119	1.4
Other	434	0.6
Vaccination dose		
First dose	48,245	61.3
Second dose	30,105	38.2
Unknown	389	0.5
Previous smallpox vaccination		
Yes	16,412	20. 8
No	54,053	68.6
Unknown	8,274	10.5
Vaccination event		
Post-exposure prophylaxis	5,467	6.9
Indication vaccination	71,347	90.6
Occupational vaccination	572	0.7
Unknown	1,353	1.7

NA: not available.

^a^ Not adjusted for the non-participating federal state.

### Individual case safety reports

The database search retrieved 32 ICSRs for Jynneos. One patient concomitantly received Symtuza, an antiretroviral drug containing darunavir, cobicistat, emtricitabine, tenofovir alafenamide, which is indicated for the treatment of infections with HIV-1. Another patient concomitantly received Comirnaty Original/Omicron BA.4-5, an mRNA COVID-19 vaccine, a third patient was concomitantly treated with risankizumab (brand name not reported), a humanised monoclonal antibody indicated for the treatment of psoriasis. Other characteristics of ICSRs are described in Table 3.

**Table 3:** Characteristic features of reported individual case safety reports after mpox vaccination, Germany, June 2022–February 2024 (n = 32)

Characteristic feature	n	%
Reporting person		
Healthcare professional	9	28.1
Patient / consumer	18	56.3
Regulatory authority	2	6.3
Pharmaceutical company	3	9.4
Gender		
Men	31	96.9
NA	1	3.1
Age (years)		
18–29	3	9.4
30–39	11	34.4
40–49	8	25.0
50–59	6	18.8
Unknown	4	12.5
Concomitant medication		
None reported	29	90.6
Symtuza	1	3.1
Comirnaty Original/Omicron BA.4-5	1	3.1
Risankizumab	1	3.1
Dose		
First dose	14	43.8
Second dose	6	18.8
Unknown	12	37.5
Seriousness^a^		
Yes, hospitalisation	3	9.4
Yes, medically important condition	4	12.5
No	25	78.1

NA: not available.

^a^ According to the ICH-E2A guideline [17].

Three Jynneos recipients were hospitalised and four ICSRs described medically important conditions. These seven ICSRs were classified as serious according to the ICH-E2A guideline [17]; details were described in Table 4. One event was assessed as "consistent with a causal association with vaccination", four as "indeterminate," one as “inconsistent with a causal association with vaccination” and seven as "unclassifiable" due to missing information.

**Table 4:** Characteristics of individual case safety reports^a^ classified as serious according to the ICH-E2A guideline [17], Germany, June 2022–February 2024 (n = 7)

Age group (years)	Report type	Cause for seriousness	Dose	Time to onset (days)	Adverse events	Causality assessment according to WHO [18]	Outcome at the time of reporting
30–39	Consumer report	Hospitalisation	1	7	Hypotonia	Unclassifiable	Recovering
					Facial paralysis^b^	Unclassifiable	Recovering
	Medically confirmed report	Other medically important condition	1	NR	Neuralgic amyotrophy of the shoulder	Unclassifiable	Not recovered
40–49	Medically confirmed report	Hospitalisation	1	17	Headache	Consistent with a causal association with vaccination	Unknown
					Ophthalmic herpes zoster	Indeterminate	Not recovered
50–59	Consumer report	Other medically important condition	1	10	Facial paresis	Indeterminate	Not recovered
	Medically confirmed report	Other medically important condition	1	10	Myelin oligodendrocyte glycoprotein antibody-associated disease^c^	Unclassifiable	Recovered
					Inappropriate route of vaccination	Unclassifiable	Recovered
	Medically confirmed report	Other medically important condition	1	14	Diarrhoea	Unclassifiable	Recovered
					Hepatomegaly	Unclassifiable	Unknown
					Hepatitis D^d^	Inconsistent with a causal association with vaccination	Unknown
Unknown	Medically confirmed report	Hospitalisation	NR	2	Visual impairment	Indeterminate	Recovering
					Bilateral optic neuritis	Indeterminate	Recovering

NR: not reported; WHO: World Health Organization.

^a^ All reports concerned males.

^b^ Acute facial peripheral facial paralysis (Bell’s palsy) is listed in the Summary of Product Characteristics for Imvanex with an unknown incidence [21].

^c^ This case report has been published elsewhere [30].

^d^ The patient had a history of chronic hepatitis B.

### Adverse events following immunisation

A total of 87 AEFIs were reported. Absolute and relative frequencies of reported AEFIs against mpox are shown in Table 5, stratified by system organ class. The most frequently reported AEFIs were headache (4.6%), pruritus (4.6%), diarrhoea (3.4%), injection site erythema (3.4%), pyrexia (3.4%), vaccination site reaction (3.4%) and urticaria (3.4%).

**Table 5:** Reported adverse events following mpox immunisation (preferred terms^a^), absolute and relative frequencies as well as adjusted reporting rates^b^ by system organ class in descending order, Germany, June 2022–February 2024 (n = 87)

MedDRA hierarchy	n	%	Adjusted reporting rate^b^
Eye disorders			
Visual impairment	1	1.1	1.1
Gastrointestinal disorders			
Diarrhoea	3	3.4	3.4
Nausea	2	2.3	2.3
Vomiting	2	2.3	2.3
Aphthous ulcer	1	1.1	1.1
Gastrointestinal disorder	1	1.1	1.1
Hypoesthesia oral	1	1.1	1.1
Oral mucosal erythema	1	1.1	1.1
General disorders and administration site conditions			
Injection site erythema	3	3.4	3.4
Pyrexia	3	3.4	3.4
Vaccination site reaction	3	3.4	3.4
Chills	2	2.3	2.3
Injection site induration	2	2.3	2.3
Malaise	2	2.3	2.3
Fatigue	1	1.1	1.1
Feeling cold	1	1.1	1.1
Influenza-like illness	1	1.1	1.1
Injection site discomfort	1	1.1	1.1
Injection site reaction	1	1.1	1.1
Injection site swelling	1	1.1	1.1
Injection site warmth	1	1.1	1.1
Local reaction	1	1.1	1.1
Pain	1	1.1	1.1
Swelling	1	1.1	1.1
Vaccination site pain	1	1.1	1.1
Vaccination site swelling	1	1.1	1.1
Hepatobiliary disorders			
Hepatomegaly	1	1.1	1.1
Infections and infestations			
Abscess	1	1.1	1.1
Hepatitis D	1	1.1	1.1
Herpes zoster	1	1.1	1.1
Nasopharyngitis	1	1.1	1.1
Ophthalmic herpes zoster	1	1.1	1.1
Oral herpes	1	1.1	1.1
Pustule	1	1.1	1.1
Injury, poisoning and procedural complications			
Incomplete course of vaccination	1	1.1	1.1
Incorrect route of product administration	1	1.1	1.1
Metabolism and nutrition disorders			
Decreased appetite	1	1.1	1.1
Musculoskeletal and connective tissue disorders			
Myalgia	2	2.3	2.3
Pain in extremity	2	2.3	2.3
Nuchal rigidity	1	1.1	1.1
Nervous system disorders			
Headache	4	4.6	4.5
Ageusia	1	1.1	1.1
Facial paralysis	1	1.1	1.1
Facial paresis	1	1.1	1.1
Hypotonia	1	1.1	1.1
Myelin oligodendrocyte glycoprotein antibody-associated disease	1	1.1	1.1
Neuralgic amyotrophy	1	1.1	1.1
Optic neuritis	1	1.1	1.1
Somnolence	1	1.1	1.1
Psychiatric disorders			
Derealisation	1	1.1	1.1
Insomnia	1	1.1	1.1
Sleep disorder	1	1.1	1.1
Skin and subcutaneous tissue disorders			
Pruritus	4	4.6	4.5
Urticaria	3	3.4	3.4
Eczema	2	2.3	2.3
Erythema	2	2.3	2.3
Rash	2	2.3	2.3
Blister	1	1.1	1.1
Pain of skin	1	1.1	1.1
Rash vesicular	1	1.1	1.1
Social circumstances			
Impaired work ability	1	1.1	1.1
Total for all AEFIs	87	100	98.7

AEFIs: adverse events following immunisation; MedDRA: Medical Dictionary for Regulatory Activities.

^a^ All listed entries here were as per coding of the adverse events reported by the notifying person and may therefore overlap.

^b^ n/100,000 doses (based on 78,739 + estimated number of vaccine doses administered in the federal state that did not participate in the voluntary mpox vaccination monitoring = 88,160 doses administered).

Adjusted reporting rates were based on an estimated total number of doses administered (88,160 doses), not only on the 78,739 doses directly reported in the monitoring system. The adjusted ICSR rate was 36.3 per 100,000 doses administered, and the adjusted serious ICSR rate was 7.9 per 100,000 doses administered. The adjusted adverse events reporting rate was 98.7 adverse events per 100,000 doses administered (1 adverse event / 1,013 doses administered).

The median number of AEFIs per ICSR was 2 (1st–3rd quartile: 1–4), median time to symptom onset was 1 day (1st–3rd quartile: 1–3). Most outcomes of AEFIs were reported as “not recovered/not resolved” (n = 35, 40.2%), “recovered/resolved” (n = 21, 24.1%) or “unknown” (n = 21, 24.1%), whereas 10 AEFIs (11.5%) were resolving at the time of reporting. There were no fatal cases. Sixty-three (72.4%) AEFIs were assessed as “consistent with a causal association to immunisation”, eight (9.2%) as “indeterminate”, one (1.1%) as “inconsistent with a causal association to immunisation” and 15 (17.2%) as “unclassifiable”.

## Discussion

The mpox vaccination campaign was rolled out after the outbreak in Germany in 2022. The surveillance of mpox cases, the mpox vaccination monitoring and the spontaneous AEFI reporting system in Germany complemented each other and helped to inform stakeholders and guide decision making. The temporal evolution of the outbreak and the demographics of mpox cases described in this paper are consistent with previous research [2,19]. The demographics of the mpox vaccine recipients mirror those of the mpox cases. Vaccination against smallpox was mandatory in the Federal Republic of Germany until 1976 and in the German Democratic Republic until 1982. In 2022, individuals who had been vaccinated against smallpox as children were aged 45 years or older in western Germany and 39 years or older in eastern Germany. Therefore, it is possible that a certain proportion of the population may have had prior immunity to mpox.

The majority of AEFIs were either local reactions (e.g. injection site erythema) or systemic reactions known to be associated with vaccination in general, such as headache, nausea, pyrexia or chills, which are consistent with the safety profiles of Jynneos [20] and Imvanex [21]. Our data demonstrate a safety profile similar to other studies of MVA-BN safety which have generally described a high incidence of local site reactions but have otherwise demonstrated that MVA-BN is well tolerated [11,22-25]. No new safety signals were identified. For one of the seven ICSRs classified as serious, causality of at least one of the reported adverse events (headache) was assessed as “consistent with a causal association with immunisation”. Acute facial peripheral facial paralysis is labelled in the summary of product characteristics for Imvanex with unknown incidence [21]. Myelin oligodendrocyte glycoprotein antibody-associated disease is a rare, antibody-mediated inflammatory demyelinating disorder of the central nervous system [26]. Hitherto, demyelinating disorders have not been associated with mpox vaccines.

Germany has a strong mandatory reporting system for infectious diseases, including mpox, and a mandatory spontaneous reporting system for AEFIs. When the first cases of mpox were reported in Germany in May 2022, stocks of Jynneos were immediately purchased from the US and a voluntary reporting system for mpox vaccinations was set up. Our work was made possible by the use of data that complied with data protection regulations and originated from these systems.

All three reporting systems are susceptible to the inherent problems of passive surveillance, such as under-reporting (mpox cases, mpox vaccinations and AEFIs), over-reporting (particularly common for drugs that are new on the market) or selective reporting (serious adverse events are more likely to be reported than non-serious adverse events). Under-reporting is particularly likely to be an issue in voluntary reporting. Information bias resulting from under-reporting, over-reporting, or selective reporting cannot be ruled out. For example, the number of vaccinations actually administered may be higher than the number reported in the voluntary vaccination surveillance system. The true reporting rate of adverse events may therefore be lower. Similarly, the true number of adverse events following mpox vaccination may be higher, which would correspond to a higher adjusted adverse events reporting rate. The extent to which these systems are complete is unknown. In addition, as the vaccination utilisation doses distributed in the participating federal states ranged from 12.5% to 60.5% with a mean vaccine utilisation of 35.5%, we might have under- or overestimated the total amount of doses administered in Germany when using the mean vaccine utilisation to calculate the number of doses administered in the one non-participating federal state. This might have led to over- or underestimation of the adjusted adverse events reporting rate.

Furthermore, the mpox vaccination campaign is not comparable to the COVID-19 vaccination campaign, in which the entire population was vaccinated, but was limited to a subsample of the population at increased risk of mpox. This does not allow the detection of AEFIs with an incidence of ≤ 1 per 100,000. So, rare adverse events or serious adverse events might have been missed, which may have led to an underestimation of the adjusted adverse events reporting rate (also for serious adverse events assessed as “consistent with a causal association with vaccination”).

In Germany, there were no reports on myocarditis/pericarditis following immunisation with smallpox vaccine until February 2024. A recent publication which extracted records of vaccine‐associated pericarditis and myocarditis between 1969 and 2023 from the WHO international pharmacovigilance database (over 130 million reports) found that smallpox vaccines were associated with most pericarditis and myocarditis reports (reporting odds ratio: 73.68; 95% confidence interval: 67.79–80.10) on the global scale [27]. However, the high reporting odds ratio in the cited global analysis was driven mainly by traditional replicating smallpox vaccines used in earlier decades. Current evidence does not suggest a similarly elevated risk for MVA-BN, although continued monitoring is warranted. Another recent publication on the safety of Jynneos utilising data from the Vaccine Safety Datalink during the 2022 mpox outbreak in the US identified no statistically significant elevated standardised incidence ratios for 10 adverse events of special interest including myocarditis/pericarditis, acute myocardial infarction, haemorrhagic stroke, ischemic stroke, venous thromboembolism, acute disseminated encephalomyelitis, encephalitis, Guillain-Barré syndrome and transverse myelitis [28].

The WHO International Pharmacovigilance Database, and systems such as the Vaccine Safety Datalink, are large enough to identify rare mpox AEFIs. It should be noted that ICSRs from Germany have been and will continue to be forwarded to both EudraVigilance and the WHO International Pharmacovigilance Database.

Regarding the outcome of an AEFI, we have reported here the outcome at the time of reporting, which in many cases is “not yet recovered/not yet resolved”. If we did not receive any follow-up information, the final outcome remained unknown.

The way in which AEFIs are reported has changed in Germany since consumers were asked in 2012 to report them themselves through a web portal. For all vaccines in 2022 and 2023 excluding those for the prevention of SARS-CoV-2 infection, most ICSRs (47.6%) originated from physicians, followed by pharmaceutical companies (27%) and patients/consumers (25.5%) [29]. This difference in reporting behaviour may be associated with the characteristics of mpox vaccine recipients; for example, mpox vaccination may be linked to MSM, which may still be a taboo topic. However, it is unclear whether this discordant reporting behaviour (the majority of persons reporting adverse events following Jynneos were patients/consumers while most ICSRs for other vaccines in Germany originate from physicians) might lead to under- or over-reporting of AEFIs.

## Conclusion

In Germany, the safety profile of Jynneos appears to be favourable in the vaccinated high-risk population, thereby supporting targeted vaccination strategies. Nevertheless, the possibility of very rare serious events cannot be discounted, and ongoing surveillance is required to monitor this.

## Data Availability

Data are available upon reasonable request.

## Supplementary Data

195000 Supplement
