# Supplementary material for: Vaccine safety surveillance during an mpox outbreak, Germany, June 2022 to February 2024
Source: Euro Surveill. 2026 Jul 30;31(30):2500843. doi: 10.2807/1560-7917.ES.2026.31.30.2500843 (PMC13428197; doi:10.2807/1560-7917.ES.2026.31.30.2500843)
Supplement: Supplement [file 25_00843_OBERLE_Supplement.pdf]

Supplementary Material

This supplementary material is hosted by *Eurosurveillance* as supporting information alongside the article “Vaccine safety surveillance during a mpox outbreak, Germany, June 2022 – February 2024”, on behalf of the authors, who remain responsible for the accuracy and appropriateness of the content. The same standards for ethics, copyright, attributions and permissions as for the article apply. Supplements are not edited by *Eurosurveillance* and the journal is not responsible for the maintenance of any links or email addresses provided therein.

Mpox reference definition, [1]

Clinical picture
Clinical picture of Mpox, defined as **at least one** of the following three criteria:
- ►Fever,
- Skin rash, skin changes (skin lesions) or mucous membrane changes (mucous membrane lesions),
- Swollen lymph nodes

OR ►Death due to the disease.

Laboratory diagnostic evidence
Positive findings using the following method:

[direct pathogen detection:]
- ►nucleic acid detection (e.g. using PCR or genome sequencing).

Clinically and laboratory-confirmed disease

Clinical picture of mpox and laboratory confirmation.

Laboratory-confirmed infection without clinical picture

Laboratory confirmation with known clinical picture that does not meet the criteria for mpox. This also includes asymptomatic infections.

Laboratory-confirmed infection with unknown clinical picture

Laboratory confirmation in the absence of information on the clinical picture (cannot be determined or not collected).

According to ICH Topic E 2 A, Clinical Safety Data Management: Definitions and Standards for Expedited Reporting: Reference Number: CPMP/ICH/377/95 [2] a serious adverse event (experience) or reaction is any untoward medical occurrence that at any dose:

- results in death,
- is life-threatening[1],
- requires inpatient hospitalisation or prolongation of existing hospitalisation,
- results in persistent or significant disability/incapacity,
- is a congenital anomaly/birth defect, or
- is an important medical event[2].

According to the WHO algorithm and classification [3] the levels of causality are:

- consistent with a causal association to immunisation,
- indeterminate,
- inconsistent with a causal association to immunisation, and
- unclassifiable.

The WHO causality assessment algorithm (according to [3]) is guided by four key questions:

1. Is there a strong evidence for other causes?
2. Is there a known causal association with the vaccine/vaccination?
3. Is there a strong evidence against a causal association?
4. Are there other qualifying factors?

---

[1] NOTE: The term "life-threatening" in the definition of "serious" refers to an event in which the patient was at risk of death at the time of the event; it does not refer to an event which hypothetically might have caused death if it were more severe.

[2] Medical and scientific judgement should be exercised in deciding whether expedited reporting is appropriate in other situations, such as **important medical events** that may not be immediately life-threatening or result in death or hospitalisation but may jeopardise the patient or may require intervention to prevent one of the other outcomes listed in the definition above. These should also usually be considered serious. Examples of such events are intensive treatment in an emergency room or at home for allergic bronchospasm; blood dyscrasias or convulsions that do not result in hospitalisation; or development of drug dependency or drug abuse.

WHO classification (according to [3])

I. Case with adequate information for causality conclusion

A case with adequate information for causality conclusion can be classified as follows:

A. Consistent causal association to immunisation
A1. Vaccine product-related reaction; or
A2. Vaccine quality defect-related reaction; or
A3. Immunisation error-related reaction; or
A4. Immunisation anxiety-related reaction/Immunisation stress related response (ISRR).

B. Indeterminate
B1. Temporal relationship is consistent but there is insufficient definitive evidence that vaccine caused the event (it may be a new vaccine-linked event). This is a potential signal and needs to be considered for further investigation.
B2. Reviewing factors result in conflicting trends of consistency and inconsistency with causal association to immunisation (i.e. it may be vaccine-associated as well as coincidental and it is not possible clearly to favour one or the other).

C. Inconsistent causal association to immunisation (coincidental)
This could be due to underlying or emerging condition(s) or conditions caused by exposure to something other than vaccine.

II. Case without adequate information for causality conclusion

Cases are categorised as "**unclassifiable**". Additional information is required for further review of causality. The available information on unclassifiable cases should be placed in a repository or an electronic database which should be periodically reviewed to see if additional information is available for classification and to perform analyses for identifying signals.

References

[1] Robert-Koch Institut. Falldefinitionen des Robert Koch-Instituts zur Übermittlung von Erkrankungs- oder Todesfällen und Nachweisen von Krankheitserregern: gemäß § 11 Abs. 2 des Gesetzes zur Verhütung und Bekämpfung von Infektionskrankheiten beim Menschen (Infektionsschutzgesetz - IfSG) [Internet]. Available from: https://www.rki.de/DE/Themen/Infektionskrankheiten/Meldewesen/Falldefinitionen/Downloads/Mpox.pdf?__blob=publicationFile&v=3. (last accessed on 7 April 2026)

[2] European Medicines Agency. ICH Topic E 2 A Clinical Safety Data Management: Definitions and Standards for Expedited Reporting: Reference Number: CPMP/ICH/377/95 Legal effective date: 01/06/1995 [Internet]. Available from: https://www.ema.europa.eu/en/documents/scientific-guideline/international-conference-harmonisation-technical-requirements-registration-pharmaceuticals-human-use-topic-e-2-clinical-safety-data-management-definitions-and-standards-expedited-reporting-step_en.pdf (last accessed on 7 April 2026).

[3] World Health Organization. Causality assessment of an adverse event following immunization (AEFI): user manual for the revised WHO classification second edition, 2019 update. [Internet]. Available from: https://iris.who.int/bitstream/handle/10665/340802/9789241516990-eng.pdf. (last accessed on 7 April 2026)
